# Alzheimer's Disease as Type 3 Diabetes: The Impact of Insulin Resistance and the Therapeutic Potential of Semaglutide in Cognitive Decline

**DOI:** 10.1002/alz70855_103762

**Published:** 2025-12-24

**Authors:** Isha Verma, Monica Kumar, Kirpal Bains, Arjun Singh, Deepesh Khanna

**Affiliations:** ^1^ Nova Southeastern University, Clearwater, FL, USA

## Abstract

**Background:**

Alzheimer's disease (AD) is a neurodegenerative disorder with limited treatments. Despite research, no cure exists, and current treatment is temporary relief. Emerging evidence links insulin resistance (IR), associated with type 2 diabetes, to AD pathology, contributing to neuroinflammation, amyloid‐β buildup, and tau hyperphosphorylation, hallmarks to AD.

**Method:**

A comprehensive literature search was conducted using PubMed and Google Scholar to identify relevant studies published between 2012 and 2024. The search employed the following keywords: “semaglutide,” “Ozempic,” “GLP‐1 receptor agonist,” “insulin resistance,” and “Alzheimer's disease.” Inclusion criteria consisted of peer‐reviewed articles, including clinical trials, meta‐analyses, and preclinical studies, that specifically examined the impact of semaglutide on insulin resistance and Alzheimer's disease. Only studies published in English and indexed in PubMed were considered for inclusion. Exclusion criteria included studies that did not address the effects of semaglutide on Alzheimer's disease, non‐peer‐reviewed articles, editorials, case reports, and studies conducted on non‐human subjects. This methodology ensured the inclusion of high‐quality, relevant studies to provide a robust analysis of semaglutide's potential role in addressing insulin resistance and neurodegeneration in Alzheimer's disease. This review synthesizes research on semaglutide's effects on IR and cognitive decline in AD.

**Result:**

Semaglutide, a GLP‐1 receptor agonist also known as Ozempic, has shown promise for AD by addressing IR‐related neurodegeneration. It enhances insulin sensitivity, reduces inflammation, and improves glucose metabolism. Importantly, it crosses the blood‐brain barrier, influencing neuronal insulin signaling and exerting potential neuroprotective effects. Studies suggest semaglutide reduces amyloid‐β, tau phosphorylation, and neuroinflammation, thus improving cognition. GLP‐1 receptor activation also enhances synaptic plasticity and neuronal survival, Early clinical studies, such as the ELAD trial, show promise for GLP‐1 receptor agonists in AD. Observational studies suggest a lower AD risk in type 2 diabetes patients using semaglutide, with a large‐scale analysis linking its use to a reduced AD incidence. The Phase 3 EVOKE trial, concluding in 2025, will further investigate semaglutide's efficacy in early AD.

**Conclusion:**

While initial evidence is encouraging, long‐term safety, optimal dosage, and impact on AD progression remain unclear. If proven effective, semaglutide could serve as a dual‐purpose drug, addressing both metabolic dysfunction and neurodegeneration.

